# Adherence to pharmacological treatment after hematopoietic stem cell transplantation: context analysis

**DOI:** 10.1590/0034-7167-2024-0265

**Published:** 2025-11-28

**Authors:** Larissa Beatriz Francisca de Souza, Katiane Domingos Soares, Allyne Fortes Vitor, Marcos Antonio Ferreira, Viviane Euzébia Pereira Santos, Isabelle Campos de Azevedo

**Affiliations:** I Universidade Federal do Rio Grande do Norte. Natal, Rio Grande do Norte, Brazil; II Universidade Federal de Mato Grosso do Sul. Campo Grande, Mato Grosso do Sul, Brazil

**Keywords:** Medication Adherence, Patient Compliance, Drug Therapy, Hematopoietic Stem Cell Transplantation, Review., Cumplimiento de la Medicación, Cooperación del Paciente, Quimioterapia, Trasplante de Células Madre Hematopoyéticas, Revisión.

## Abstract

**Objectives:**

to analyze the contexts that influence adherence to pharmacological treatment after hospital discharge in patients in the post-hematopoietic stem cell transplantation phase.

**Methods:**

context analysis based on the framework proposed by Hinds, Chaves, and Cypress, operationalized through a scoping review, in which 27 studies were selected to support the discussion.

**Results:**

the data were grouped at each contextual level according to their particularities and are presented under the following subthemes: psychological, physical, and cognitive aspects of the patient; facilitators and barriers to medication management at home; particularities of the patient-professional relationship; institutional guidelines and programs for the care of patients after hematopoietic stem cell transplantation.

**Final Considerations:**

the contextual elements highlight the need to ensure holistic care, with the engagement of professionals, patients, and family members, as well as to recognize poor medication adherence as a potential public health issue.

## RESUMEN

**Objetivos:**

analizar los contextos que influyen en la adhesión al tratamiento farmacológico tras el alta hospitalaria de pacientes en fase de postrasplante de células madre hematopoyéticas.

**Métodos:**

análisis de contexto de acuerdo con el marco propuesto por Hinds, Chaves y Cypress, operacionalizado mediante una revisión de alcance, en la cual se seleccionaron 27 estudios que fundamentaron la discusión.

**Resultados:**

los datos se agruparon en cada nivel contextual según sus particularidades, presentándose en subtemas, a saber: aspectos psicológicos, físicos y cognitivos del paciente; facilitadores y barreras para la gestión de la medicación en el hogar; particularidades de la relación paciente-profesional; directrices y programas institucionales para el cuidado del paciente tras el trasplante de células madre hematopoyéticas.

**Consideraciones Finales:**

los elementos contextuales destacan la necesidad de garantizar una atención holística, con la participación de profesionales, pacientes y familiares, así como reconocer la falta de adhesión medicamentosa como un potencial problema de salud pública.

## INTRODUCTION

Adherence to pharmacological treatment is defined as the process by which patients take medications as prescribed and directed by healthcare professionals. It consists of three phases: initiation, when the patient begins taking a prescribed medication; implementation, meaning the degree to which the actual dosage matches the prescription; and persistence, when the patient continues treatment with the prescribed dosage^(1,2)^.

In the context of post-hematopoietic stem cell transplantation (HSCT), adherence to pharmacological treatment is particularly crucial, as any lack of commitment or improper administration of medications can trigger serious complications and adverse events that may require hospital readmissions^(3,4)^. Furthermore, patients with hematologic cancer face an increased risk of infections due to immunosuppression, making poor adherence to pharmacological treatment even more concerning^(5)^.

This type of transplantation is widely used as a form of cellular immunotherapy to treat malignant and benign conditions that are potentially curable^(6)^. Hospital discharge occurs when engraftment is successful, but outpatient follow-up continues for an extended period, generally encompassing the first 100 days post-HSCT^(7)^. Thus, patients are discharged with several new medications to be administered at home^(4)^.

However, the transition to outpatient follow-up after hospital discharge is a critical moment, as medication adherence tends to decline, especially due to polypharmacy and the complexity of therapeutic regimens^(8,9)^. Studies indicate that adherence rates vary from 18.8% to 100%, with immunosuppressants showing even lower rates, ranging from 31.3% to 88.8%^(8-10)^. Factors such as younger age, psychosocial distress, social support, and therapy complexity influence this adherence^(8-10)^.

It should also be noted that, despite the existence of various technologies aimed at supporting medication adherence, many of them face significant challenges regarding effective implementation and sustainable use. This likely occurs due to the lack of active involvement of end users in the development process, the scarcity of solid theoretical foundations, and insufficient attention from healthcare professionals to implementation methods that ensure effectiveness in everyday settings^(2)^.

Finally, a recent systematic review identified a scarcity of studies investigating how contextual factors that influence adherence interrelate and impact treatment continuity in the post-HSCT phase^(10)^. Thus, the present study is justified by the innovative context analysis proposed, which involves the use of an interactive layered structure to achieve a broad and comprehensive understanding of the phenomenon. It is believed that this approach will enable the development of more assertive actions to minimize adherence challenges and improve the quality of life of post-HSCT patients.

## OBJECTIVES

To analyze the contexts that influence adherence to pharmacological treatment after hospital discharge in patients in the post-HSCT phase.

## METHODS

### Theoretical and methodological framework

This is a context analysis based on the proposal by Hinds, Chaves, and Cypress (1992), which aims to elucidate the full meaning and understand the entirety of a situation or event, allowing for a comprehensive explanation of the phenomenon in question.

Context is defined as a structure composed of four interactive layers, namely: immediate, specific, general, and metacontext. These layers are distinguished from one another by three aspects: how broadly the meaning is shared, ranging from individual to almost universal meaning; the time frame on which it is focused, whether in the present or the future; and the speed at which change occurs and how it is perceived within each of these layers^(11)^.

From this perspective, the immediate context involves understanding the phenomenon as it occurs. The specific context is shaped by various circumstances of the moment in question, such as personal, environmental, and emotional factors. In the general context, interpretations developed by the individual through past and present interactions are considered. Finally, the metacontext is a source of socially constructed knowledge that directly influences behaviors and events^(11)^.

### Methodological procedures

To operationalize the context analysis, a scoping review was conducted in accordance with JBI guidelines^(12)^, following the recommendations of the Preferred Reporting Items for Systematic Reviews and Meta-Analyses extension for Scoping Reviews (Prisma-ScR), with the research protocol registered on the Open Science Framework (OSF). This method aims to map the available evidence in a specific area, identify existing gaps, and inform the need for future investigations. The choice of this type of review for the present study was intended to clarify the studied concept and identify its main characteristics or related factors, in accordance with JBI guidelines^(12)^.

The steps proposed by Peters et al.^(13)^ were followed: (1) Definition and alignment of the objective and research question; (2) Development and alignment of inclusion criteria with the objective and research question; (3) Description of the planned approach for evidence search, selection, data extraction, and presentation of the evidence; (4) Searching for evidence; (5) Selecting the evidence; (6) Extracting the evidence; (7) Analyzing the evidence; (8) Presenting the results; (9) Summarizing the evidence in relation to the review’s purpose.

To formulate the research question, the PCC mnemonic was used (P = Population: Patients in the post-HSCT phase; C = Concept: Adherence to pharmacological treatment; C = Context: Hospital discharge). Thus, the following research question was established: “What contexts influence adherence to pharmacological treatment after hospital discharge in patients in the post-HSCT phase?”.

Initially, a broad search was conducted on the OSF platform and in databases to identify protocols or reviews on a similar topic. In the absence of similar studies, the subsequent steps were carried out.

The search strategy was divided into two phases. The first consisted of an initial search in the National Library of Medicine (PubMed) and the Cumulative Index to Nursing and Allied Health Literature (CINAHL) databases to analyze the words contained in the titles and abstracts of the retrieved articles that represented the object of study. These words were searched in Medical Subject Headings (MeSH)/*Descritores em Ciências da Saúde* (DeCS), and the controlled descriptors were selected as follows: medication adherence / *adesão à medicação*; hematopoietic stem cell transplantation / *transplante de células-tronco hematopoéticas;* stem cell transplantation / *transplante de células-tronc*o; bone marrow transplantation / *transplante de medula óssea*. The following keywords were also selected: drug adherence / *adesão ao medicamento* and adherence / *adesão*.

The second phase consisted of a subsequent search in all selected data sources using the following strategy: ((“medication adherence” OR “drug adherence” OR adherence) AND (“hematopoietic stem cell transplantation” OR “stem cell transplantation” OR “bone marrow transplantation”)). It is noteworthy that, when necessary, the employed strategy was adjusted to meet the specific requirements of each data source.

### Data sources

For a comprehensive mapping of the literature on the studied topic, the following databases were selected: Scopus, Web of Science (WoS), National Library of Medicine (PubMed), CINAHL, ScienceDirect, *Literatura Latino-Americana e do Caribe em Ciências da Saúde* (LILACS), The Education Resources Information Center (ERIC), Academic Archive Online (DIVA), Repositório Científico de Acesso Aberto de Portugal (RCAAP), Theses Canada, and the CAPES database of theses and dissertations. The choice of these data sources is justified by their ability to provide access to a variety of high-relevance and high-quality scientific evidence, including primary and secondary studies, theses and dissertations, as well as national and international literature. The diversity of data sources is crucial to ensuring comprehensive and representative coverage of the research field^(12)^.

### Data collection and organization

The evidence selection stage was conducted between October and December 2023, carried out independently by two reviewers who were properly trained regarding the selection criteria for this study. This process followed two distinct stages: an initial screening, in which titles and abstracts were evaluated, resulting in the selection of studies for the second screening, where the full texts were analyzed. In case of disagreement, a third reviewer was consulted.

For the export, organization, and initial screening of the retrieved references, in addition to the identification and removal of duplicates, the software Rayyan^®^, a free computational tool developed by the Qatar Foundation, was used^(14)^.

The inclusion criteria were: studies related to adherence to pharmacological treatment during the post-HSCT phase with patients of any age and both sexes, available in full text and in any language through access via the *Comunidade Acadêmica Federada* (CAFe), without time restrictions. Editorials, letters to the editor, opinion articles, protocols, books, and book chapters were excluded. Duplicate studies were considered only once.

The data extraction strategy consisted of collecting the following variables: title, authorship, year of publication, country where the study was conducted, methodological design, objectives, results, and description of the main findings related to adherence to pharmacological treatment in the post-HSCT phase. For this purpose, a spreadsheet was used in Microsoft Excel^®^ software.

### Data analysis

The context analysis model, according to the adopted theoretical and methodological framework, was used. Thus, after the selection and full reading of the studies, the main findings related to adherence to pharmacological treatment in the post-HSCT phase were grouped based on their similarities. These influences were then categorized according to the contextual focus they represented: immediate, specific, general, or metacontext. This analysis allowed interaction among discussions to promote a comprehensive understanding. The other data extracted from the selected studies were analyzed descriptively.

## RESULTS

**Figure 1:**
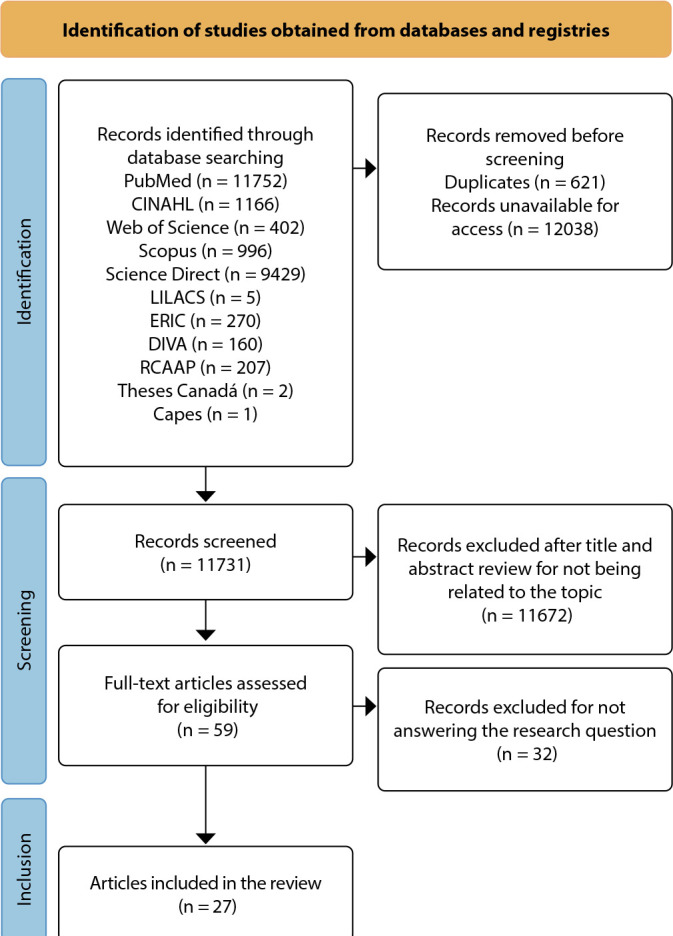
Flowchart of the study selection process, 2024

A total of 27 studies were included in the sample^(4,5,8,10,15-37)^. Figure 1 presents the flowchart of the evidence search and selection process, including the identification, screening, and inclusion of the studies.

Among the studies identified, all were articles, most of which were published in 2023, totaling seven (25.9%). The United States stood out in terms of quantity, with 10 studies (37%), followed by Germany, Brazil, and France with three studies each (11.1%). Regarding the type of methodological approach, 12 observational studies prevailed (44.4%). Chart 1 presents the other characteristics of the studies included in the sample.

**Chart 1:** Characterization of the studies included in the sample according to title, year, country, design/number of participants, interventions, and outcomes (N=27), 2024.

Title	Year / Country	Study Design/Number of Participants	Interventions	Outcomes
Evaluation of a patient self-medication program in allogeneic hematopoietic stem cell transplantation^(4)^	2022 Canada	Prospective preand post-cohort comparison n=51	Patient Self-Medication Program for patients vs. conventional discharge medication education.	Median self-efficacy scores were 38/39 in the conventional education group vs. 39/39 in the Self-Medication Program group.
Evaluation of adherence and clinical outcomes in patients undergoing allogeneic haematopoietic stem cell transplantation^(5)^	2020 Spain	Observational study n=46	Pharmacy Service dispensing records.	High adherence to prophylactic treatment, ranging from 80.4% to 100.0%.
Improving the transition of highly complex patients into the community: impact of a pharmacist in an allogeneic stem cell transplant (SCT) outpatient clinic^(8)^	2013 Australia	Prospective cohort study n=23	Consultations by a specialized clinical pharmacist.	Contribution to improving medication management and adherence.
Medication Adherence among Allogeneic Haematopoietic Stem Cell Transplant Recipients: A Systematic Review^(10)^	2023 Italy	Systematic review	Subjective adherence measures were most frequently used (78.6%) up to now.	Median prevalence of medication adherence was 61.8% and did not decrease over time. Non-adherence is multifactorial and requires multidisciplinary care models.
Reciprocal associations between beliefs about medicines, health locus of control and adherence to immunosuppressive medication in allogeneic hematopoietic cell transplant patients: Findings from the ADE-TRAM study^(15)^	2023 Italy	Observational study n=50	Adherence measured by the Immunosuppressive Medication Self-Management Scale.	Patients who believe their disease is caused by external factors are less likely to adhere to treatment.
Medication Adherence in Patients with Hematologic Malignancies Who Are Hematopoietic Stem Cell Transplantation Survivors: A Qualitative Study^(16)^	2023 United States of America	Qualitative and partially prospective longitudinal study n=30	Interviews exploring physical, social, psychological, and sociodemographic factors influencing post-transplant medication adherence.	Facilitators and barriers to medication adherence can be physical, psychological, organizational, and social.
Recommendations for Providing Medication Adherence Support After Pediatric Hematopoietic Stem Cell Transplant: Caregivers’ Lived Experience^(17)^	2023 United States of America	Qualitative study n=29	Qualitative semi-structured interviews about caregiver experience and recommendations.	Practical supports for medication adherence should be tailored based on the family’s individual needs.
Adherence to Immunosuppressants among Adult Patients after Allogeneic Hematopoietic Stem-Cell Transplantation (Allo-HSCT): A Cross-Sectional Study^(18)^	2023 Iran	Cross-sectional study n=110	Use of the validated Persian version of the 8-item Morisky Medication Adherence Scale (MMAS-8).	High rates of non-adherence, indicating the need for more careful medication use monitoring.
*Orientações para o autocuidado de pacientes no pós-transplante de células-tronco hematopoéticas: revisão de escopo* ^(19)^	2023 Brazil	Scoping review	To map the evidence on the guidelines provided for the self-care of patients in the post-HSCT phase.	Following medication-related care is one of the greatest challenges. This is related to non-adherence to pharmacotherapy, the deficit of social support, and the need for instrumental support.
Impact of the insertion of the clinical pharmacist in the Allogeneic Hematopoietic Stem Cells Transplantation team^(20)^	2023 Brazil	Intervention study n=61	Pharmacotherapeutic follow-up.	Increased knowledge and adherence.
Can pharmacotherapeutic follow-up after allogeneic hematopoietic stem cell transplantation improve medication compliance?^(21)^	2023 Brazil	Intervention study n=27	Pharmacotherapeutic follow-up.	Improvement in medication adherence and knowledge between the first and last consultations.
Medication discharge teaching in pediatric hematopoietic stem cell transplantation: teaching characteristics, caregiver perceptions, and postdischarge adherence^(22)^	2022 United States of America	Qualitative study n=19	Pharmacist-led video-recorded medication discharge teachings.	Caregivers reported high confidence in understanding and managing their child’s medication regimen.
Rates and Predictors of Nonadherence to the Post-Allogeneic Hematopoietic Cell Transplantation Medical Regimen in Patients and Caregivers^(23)^	2022 United States of America	Prospective study n=183	Modified Health Habits Assessment.	11.2-15.7% reported non-adherence to immunosuppressants, 34.8-38.6% to other medications; patient perceptions were consistent predictors.
Exploring Stem Cell Transplanted Patients’ Perspectives on Medication Self-Management and Electronic Monitoring Devices Measuring Medication Adherence: A Qualitative Sub-Study of the Swiss SMILe Implementation Science Project^(24)^	2022 Switzerland	Exploratory and qualitative substudy n=6	Integrated Care Model in allogeneic stem cell transplantation facilitated by eHealth	Participants considered the MEMS Button a viable device for monitoring medication adherence in everyday life.
Immunosuppression medication adherence after allogeneic hematopoietic stem cell transplant: Impact of a specialized clinical pharmacy program^(25)^	2021 France	Prospective interventional study n=61	Specialized clinical pharmacy program.	Serum levels within the therapeutic target range were higher in the intervention group (61.5% versus 53.0%).
“This Graft-vs.-Host Disease Determines My Life. That’s It.”-A Qualitative Analysis of the Experiences and Needs of Allogenic Hematopoietic Stem Cells Transplantation Survivors in Germany^(26)^	2021 Germany	Qualitative study n=40	Semi-structured interviews.	Participants reported non-adherence to prescribed care, such as medication or isolation, as they were overwhelmed by the myriad of instructions.
Psychosocial Pre-Transplant Screening With the Transplant Evaluation Rating Scale Contributes to Prediction of Survival After Hematopoietic Stem Cell Transplantation^(27)^	2021 Germany	Prospective study n=61	TERS (Transplant Evaluation Rating Scale) and MESI (Medication Experience Scale for Immunosuppressants) screenings.	The mean adherence score of all 61 patients assessed by attending physicians was 2/5. Patient adherence correlated significantly with TERS scores.
Medication non-adherence after allogeneic hematopoietic cell transplantation in adult and pediatric recipients: a cross sectional study conducted by the Francophone Society of Bone Marrow Transplantation and Cellular Therapy^(28)^	2021 France	Multicenter cross-sectional study n=242	Compliance Assessment Test	Age as the only factor related to non-adherence.
Clinicians and patients perspectives on follow-up care and eHealth support after allogeneic hematopoietic stem cell transplantation: A mixed-methods contextual analysis as part of the SMILe study^(29)^	2020 Germany	Mixed-methods study n=60	Chronic Care Model enhanced by eHealth; Focus groups.	Health behaviors that would benefit from support include medication adherence, physical activity, and infection prevention.
A Prospective Survey of Outpatient Medication Adherence in Adult Allogeneic Hematopoietic Stem Cell Transplantation Patients^(30)^	2020 United States of America	Cross-sectional study n=200	Morisky Medication Adherence Scale and Immunosuppressive Therapy Adherence Scale.	Half (51%; 102 out of 200) of allogeneic transplant recipients reported non-adherence to non-immunosuppressive medications.
Standardized Semi-structured Psychosocial Evaluation before Hematopoietic Stem Cell Transplantation Predicts Patient Adherence to Post-Transplant Regimen^(31)^	2019 United States of America	Retrospective study n=85	Application of the Stanford Integrated Psychosocial Assessment for Transplantation	The quantified psychosocial risk correlated with adherence to the post-transplant regimen.
Medication adherence after pediatric allogeneic stem cell transplantation: Barriers and facilitators^(32)^	2018 France	Qualitative research n=15	Semi-structured interviews were conducted by a pharmacist.	Caregivers’ need: to consider the family unit.
Facilitators and Barriers to Self-Management for Adolescents and Young Adults Following a Hematopoietic Stem Cell Transplant^(33)^	2017 United States of America	Grounded theory research n=30	Semi-structured interview.	Facilitators included having a positive attitude, social support, organization, motivation, and information.
Relationship between neurocognitive functioning and medication management ability over the first 6 months following allogeneic stem cell transplantation^(34)^	2016 Canada	Prospective study n=58	The Medication Management Task (revised) and neurocognitive functioning tests were used.	Patients with neurocognitive impairment were more likely to have impaired medication management ability in a simulated task.
Adherence to outpatient oral medication regimens in adolescent hematopoietic stem cell transplant recipients^(35)^	2014 United States of America	Intervention study n=6	Electronic pill bottles (Medical Event Monitors).	Adherence difficulties decreased from 91% during the first month after discharge to less than 80% after 3 months and less than 60% after 6 months.
Utilization of collaborative practice agreements between physicians and pharmacists as a mechanism to increase capacity to care for hematopoietic stem cell transplant recipients^(36)^	2013 United States of America	Review	Use of pharmacists to manage drug therapy through collaborative practice agreements.	Pharmacists have the knowledge base and skills to assist physicians and advanced practice providers with medication therapy management.
Medication Adherence in Hematopoietic Stem Cell Transplantation: A Review of the Literature^(37)^	2017 United States of America	Literature review	The methods used to measure adherence were based on self-report.	Adherence declined over time in all studies except in one intervention study.

Chart 2 presents a synthesis of the main findings related to adherence to pharmacological treatment in the HSCT phase identified in the sample, along with the grouping carried out according to the axis of discussion and context of analysis, following the method proposed by Hinds, Chaves, and Cypress (1992).

**Chart 2:** Synthesis of the main findings related to adherence to pharmacological treatment in the post-hematopoietic stem cell transplantation phase identified in the sample (N=27), 2024

Axis of Discussion/Context of Analysis	Main findings related to adherence to pharmacological treatment after HSCT
Intrinsic patient factors, such as psychological, physical, and cognitive aspects/Immediate context	Psychological aspects such as stress, depression, social isolation, tension, lack of motivation and perseverance, forgetfulness, and moderate to severe distress interfere with treatment adherence^(20,23,27-31)^.
	Negligence with medication due to worsening symptoms^(30)^.
	Patients with neurocognitive impairment were more likely to have impaired medication management ability^(34)^.
	Patients with less concern about immunosuppressive medications showed a greater probability of adherence^(15)^.
Medication management at home/Specific context	Facilitators for medication management: Previous experience with medications^(16)^ Support from communication materials^(17)^ Interventions: health education, medication organizers, simplification of the medication regimen, reminders, behavior modification, social support, support and follow-up by healthcare providers, and insurance incentives^(8,22,26,33,37)^.
	Barriers to medication management: Difficulty in medication supply and insurance logistical management^(16)^ New conditions at home^(18)^ Regarding treatment: medication dosing time, complexity of the treatment, and frequent medication changes^(16,21,33)^ Information overload^(26)^ Type of transplant performed^(31,36)^
Relationship between professionals and patients/General context	Lack of adequate patient education regarding medication use interferes with adherence^(18)^.
	Post-HSCT patients should be aware of self-care guidelines, enabling the nursing team to provide the necessary information for home care^(19)^.
	Nursing supervision in patients’ self-administration of medications^(10)^.
	Need for good interactions between patient, caregiver, and healthcare system to enable and empower patients to follow treatment^(33)^.
	Some patients may require intensive intervention efforts before and immediately after discharge^(36)^.
Institutional guidelines and programs/Metacontext	The Patient Self-Medication Program is associated with long-lasting and improved medication knowledge^(4)^.
	Guidelines from the National Marrow Donor Program^(23)^.
	Deficits exist in the hospital transplant program regarding discharge preparation, support for self-management, and transitional care^(24)^.
	Specialized Clinical Pharmacy Program can be beneficial for adherence to immunosuppressive drugs^(25)^.

After performing the grouping and categorization by contextual focus, the following subthemes were created:

- Immediate context: psychological, physical, and cognitive aspects of the patient;
- Specific context: facilitators and barriers to medication management at home;
- General context: particularities of the patient-professional relationship;
- Metacontext: institutional guidelines and programs for post-HSCT patient care.

**Figure 2:**
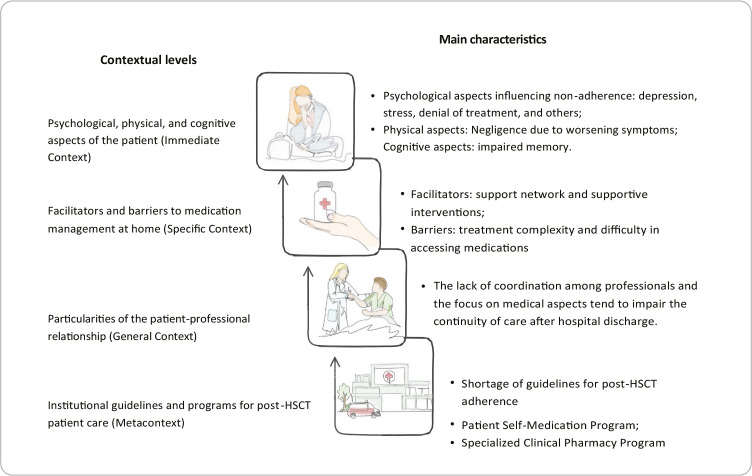
Contextual layers of adherence to pharmacological treatment in the post-hematopoietic stem cell transplantation phase, 2024

Additionally, a representation of the interrelated layers and their main characteristics was created, as shown in Figure 2.

## DISCUSSION

### Immediate context: psychological, physical, and cognitive aspects of the patient

The effectiveness of HSCT is intrinsically linked to strict adherence to drug therapy, which often requires the administration of multiple medications at specific times and dosages. However, maintaining this adherence in the long term can become a substantial challenge, mainly due to patient-associated factors. These include psychological, physical, and cognitive aspects that directly impact the patient’s ability to follow the prescribed treatment, either intentionally or unintentionally^(20,23,27-31,34)^.

Among these, psychological distress stood out the most, negatively affecting healthcare outcomes. Depression, stress, social isolation, psychosocial tension, anguish, and denial of treatment were the most frequently reported aspects^(15,20,27-29,31)^. A possible reason for these findings is concern about the transplant prognosis and the potential complications during the recovery phase^(38)^.

This finding strengthens the conclusions of a recent study indicating that subjective perceptions about health status and medication use may be more relevant in explaining adherence behavior than non-modifiable characteristics such as age and sex^(39)^.

Moreover, individual coping strategies also influence patient cooperation with the prescribed treatment. Given the variability of coping strategies among individuals, it becomes difficult to define an ideal type of adaptation during the post-transplant period; however, it is crucial to emphasize that when poorly managed, these strategies can cause negative and harmful effects, such as the emergence of anxiety crises, depression, and lower treatment adherence^(16,39)^.

Physical aspects are related to medication side effects, such as nausea, fatigue, and other discomforts. These symptoms may vary in intensity and frequency among patients, affecting their motivation and willingness to adhere to the treatment. Furthermore, the transplant process itself can be extremely physically exhausting, making it even more challenging for individuals to remain committed to prescribed therapies^(17)^.

At such times, it is natural for patients to question the real effectiveness of the treatment and whether the discomforts are bearable, especially considering the long recovery period after transplantation. In this context, it is observed that physical and psychological factors interact in a complex manner, as they may contribute to the trivialization of the treatment and a lack of motivation^(2)^.

Additionally, patients with cognitive impairment may experience difficulty remembering the instructions regarding pharmacological treatment, resulting in impaired medication management^(30,34)^. Although it is a specific challenge, it is important to highlight the relationship between subjective cognitive complaints and psychological distress, particularly when facing conflicting situations. Thus, it can be suggested that neuropsychological performance may not be the main reason for the reported cognitive problems^(40)^.

### Specific context: facilitators and barriers to medication management at home

The management of pharmacological treatment after hospital discharge refers to the measures and practices adopted to ensure that patients continue taking prescribed medications appropriately at home. Support networks and supportive interventions are considered facilitators of this management^(16,17)^.

The inclusion of other people in a support network, such as family members and friends, is essential for patient-centered care and is considered the main facilitator of therapeutic adherence. For this support to be effective, it is necessary to encourage the patient’s independence and autonomy without confusing support with control over daily activities^(41)^.

In this context, the role of support may include coping with long-term transplant sequelae, assisting in recalling information provided by healthcare professionals, and providing guidance in managing post-discharge care^(32,37)^. In addition to social support, supportive interventions have also been shown to facilitate adherence. Examples include the use of medication organizers, phone alarms, and educational manuals, whose main objective is to minimize confusion and forgetfulness^(16,20,37)^.

According to a systematic review, understanding the purpose and correct use of medication is fundamental for individuals to take responsibility for their treatment, becoming engaged participants in the prescription and medication administration process^(42)^. Thus, interventions should be incorporated into a broader educational context, meaning they should complement the guidance and other care provided by the healthcare team^(21)^.

On the other hand, with advances in treatment modalities, survival rates after transplantation have increased significantly. However, this scenario has also brought the challenge of self-management of prescribed medications over a long post-transplant period. As a result, the very nature of the therapy has become the main barrier mentioned in the studies included in the review^(5,37)^. The impact of this challenge may vary depending on the type of transplant performed. For example, in the case of autologous transplantation compared to allogeneic transplantation, there is generally a shorter hospitalization period, less intensive follow-up, and no long-term prescription of immunosuppressants^(31)^.

Difficulty in accessing medications was also identified as another barrier, partially due to the logistical management of health insurance and delayed medication refills by specialized pharmacies^(16)^. A meta-analysis revealed that there is an 11.0% higher probability of non-adherence among populations with health insurance that requires copayment for medication purchases. This lack of adherence can increase healthcare system costs due to the greater expenses associated with hospitalizations caused by the non-use of essential medications^(43)^.

### General context: particularities of the patient-professional relationship

The specificities surrounding the patient-professional relationship directly influence treatment adherence, either by creating obstacles or facilitating the self-care process. It is important to understand that this relationship goes beyond medical consultations, as it also involves other professionals, especially the nursing team, and includes the approach to self-care for patients undergoing HSCT^(19)^.

Regarding the healthcare team, it was observed that a lack of coordination among professionals contributed to greater confusion regarding the guidance provided to patients and their families^(32)^. This fact can be considered a reflection of the Flexnerian paradigm that shaped the curricular matrices of undergraduate health courses, which emphasized biological and medicalized aspects without an integrated view of the patient as a whole^(42)^.

It is evident that this fragmented approach may impair the quality and continuity of patient care at home. Thus, positive communication within the healthcare team becomes a valuable ally in promoting better adherence to treatment. In addition, adopting an approach sensitive to the uniqueness of each individual is a strategy capable of enhancing the patient’s self-management of care after transplantation^(44)^.

Moreover, an excessive focus on medical aspects has been identified as another obstacle in this relationship, meaning a predominant emphasis on pharmacological therapy and disease cure, sometimes to the detriment of attention to patient self-management of care. In fact, new medical technologies have facilitated higher chances of cure but do not always alleviate the suffering of patients dealing with life-limiting diseases^(45)^.

Furthermore, considering psychological distress as a factor for non-adherence to treatment, it becomes necessary for the care of the transplanted patient to transcend a curative approach. It is essential that the healthcare team know how to address patients’ concerns and fears, encouraging them to express these in relation to their treatment. The redefinition of post-HSCT care may contribute to greater acceptance of treatment, as patients come to understand the reasons behind such care^(46)^.

### Metacontext: institutional guidelines and programs for post-HSCT patient care

The traditional medical model assumes that the effectiveness of treatment is sufficient to guarantee patient adherence, which may neglect other important factors that influence treatment adherence^(39)^. Due to the hegemony of this healthcare paradigm, the guidelines found in the literature have focused on healthcare professionals, and a gap has been noted in research dedicated to the best recommendations regarding post-HSCT adherence^(31,47)^.

It is worth noting that patients often do not follow self-care instructions due to a lack of understanding of the importance of these guidelines for treatment^(19)^. Research conducted with patients in Brazil and Spain reveals that the amount of information included in discharge instructions often leaves transplant patients with doubts and uncertainties about the necessary care at home. Additionally, some participants explicitly mentioned that they did not follow important instructions, such as the use of sunscreen, condoms during sexual intercourse, and dietary precautions^(48)^.

As a consequence, a disconnection between hospital and outpatient care is observed, resulting in failures in communication, inadequate information exchange, and impaired monitoring after hospital discharge^(24,29)^. This situation could be mitigated through investments in infrastructure and team training to adopt a more coordinated approach to care, along with the implementation of integrated care programs.

In this regard, two studies in the present sample evaluated the impact of health programs for post-HSCT patients. One of them is the Patient Self-Medication Program implemented in transplant units in Canada, where patients with scheduled hospital discharge receive medications in labeled vials, similar to those available at conventional pharmacies, allowing for self-administration supervised by a nurse^(4)^.

The study concluded that this program was associated with better knowledge about medication after discharge, persisting for three to five weeks compared to previous care. The strategy used is based on the assumption that knowledge is particularly vital in this population, given that the complexity of the medication regimen can cause confusion and, consequently, affect adherence^(4)^.

The other is the Specialized Clinical Pharmacy Program implemented at a hospital institution in France. It was based on pharmaceutical consultations conducted one day before hospital discharge, at two and four weeks after discharge, and then once a month until completing 100 days post-HSCT. During the consultation, the patient received guidance regarding the medication prescription, reinforcing the importance of adherence and providing a personalized schedule for medication administration^(25)^.

Thus, it is essential that the multiprofessional team, especially the nurse, implement effective educational actions to ensure that the instructions on care and self-care to be followed at home after hospital discharge are properly understood and applied. The nurse plays a crucial role in this process due to their proximity to the patient, their ongoing educational role, and their ability to provide detailed clarifications, facilitating adherence to the instructions and improving the effectiveness of home care^(48)^.

### Study limitations

As a limitation of the study, the scarcity of intervention studies in the sample is highlighted, which could provide a more robust understanding of the impact of specific contexts on adherence to pharmacotherapy.

### Contributions to the Field of Nursing, Health, or Public Policy

The description of the different contexts that influence adherence to pharmacological treatment after HSCT not only expands theoretical knowledge in nursing but also provides professionals with evidence-based guidance for building interventions that support and assist treatment. The study also offers an understanding of the patient experience, facilitating comprehensive healthcare with a focus on patient needs. With these guidelines, it is expected that the quality of post-HSCT care will be improved, encouraging the development of further research in the field to broaden the discussion even more.

## FINAL CONSIDERATIONS

Adherence to pharmacological treatment after HSCT is influenced by different interrelated contextual factors, manifested through the complex interaction among each patient’s physical, psychological, cognitive, and economic influences, the quality of the relationship established with healthcare professionals, and the presence of external facilitators and barriers, which are not always modifiable. Additionally, the global context, referring to healthcare guidelines and programs, plays a crucial role in supporting and ensuring the overall effectiveness of the treatment.

There is a need for comprehensive research on adherence to pharmacological treatment after HSCT in Brazil, especially given the country’s cultural, socioeconomic, and healthcare access diversity, which may significantly influence adherence. For this purpose, multicenter studies are essential, as they allow for a more representative approach by including participants from different regions of the country.

## Data Availability

Not applicable.
