# Supplementary material for: Adherence to pharmacological treatment after hematopoietic stem cell transplantation: context analysis
Source: Rev Bras Enferm. 2025 Nov 28;78(5):e20240265. doi: 10.1590/0034-7167-2024-0265 (PMC12673612; doi:10.1590/0034-7167-2024-0265)
Supplement: Translation

# Translation: Adesão ao tratamento farmacológico no pós-transplante de células-tronco hematopoéticas: análise de contexto

**DOI:** 10.1590/0034-7167-2024-0265pt

**Authors:** Larissa Beatriz Francisca de Souza, Katiane Domingos Soares, Allyne Fortes Vitor, Marcos Antonio Ferreira, Viviane Euzébia Pereira Santos, Isabelle Campos de Azevedo

**Affiliations:** I Universidade Federal do Rio Grande do Norte. Natal, Rio Grande do Norte, Brasil; II Universidade Federal de Mato Grosso do Sul. Campo Grande, Mato Grosso do Sul, Brasil

**Keywords:** Adesão à, Medicação, Cooperação do Paciente, Tratamento Farmacológico, Transplante de Células-Tronco Hematopoéticas, Revisão.

## Abstract

**Objetivos:**

analisar os contextos que influenciam a adesão ao tratamento farmacológico após a alta hospitalar de pacientes em fase de pós-transplante de células-tronco hematopoéticas.

**Métodos:**

análise de contexto de acordo com o referencial proposto por Hinds, Chaves e Cypress, operacionalizada por uma revisão de escopo, no qual foram selecionados 27 estudos que subsidiaram a discussão.

**Resultados:**

os dados foram agrupados em cada nível contextual de acordo com suas particularidades, apresentando-se em subtemas, a saber: aspectos psicológicos, físicos e cognitivos do paciente; facilitadores e barreiras para o gerenciamento da medicação em domicílio; particularidades da relação paciente-profissional; diretrizes e programas institucionais para o cuidado do paciente pós-transplante de células-tronco hematopoéticas.

**Considerações Finais:**

os elementos contextuais ressaltam a necessidade de garantir um cuidado holístico, com envolvimento entre profissionais, pacientes e familiares, bem como reconhecer a falta de adesão medicamentosa como um potencial problema de saúde pública.

## INTRODUÇÃO

A adesão ao tratamento farmacológico é definida como o processo pelo qual os pacientes utilizam medicamentos conforme prescrição e orientação de profissionais de saúde. Além disso, é composto por três fases: início, quando o paciente começa a tomar uma medicação prescrita; implementação, que significa a correspondência da dosagem real em comparação com a prescrição; e persistência, quando o paciente dá continuidade ao tratamento com a dose real prescrita^(1,2)^.

No contexto do pós-transplante de células-tronco hematopoéticas (TCTH), a adesão ao tratamento farmacológico é particularmente crucial, pois qualquer falta de comprometimento ou administração inadequada dos medicamentos pode desencadear complicações graves e eventos adversos que podem exigir readmissões hospitalares^(3,4)^. Ademais, pacientes com câncer hematológico enfrentam risco aumentado de infecções devido à imunossupressão, o que torna a baixa adesão ao tratamento farmacológico ainda mais preocupante^(5)^.

Esse tipo de transplante é amplamente usado como uma forma de imunoterapia celular para tratar condições malignas e benignas potencialmente curáveis^(6)^. A alta hospitalar ocorre quando o enxerto é bem-sucedido, mas o acompanhamento ambulatorial persiste por um período longo, geralmente durante os primeiros 100 dias pós-TCTH^(7)^. Assim, os pacientes recebem alta com vários novos medicamentos a serem administrados em domicílio^(4)^.

A transição para o acompanhamento ambulatorial após a alta hospitalar é, portanto, um momento crítico, pois a adesão aos medicamentos tende a diminuir, especialmente devido à polifarmácia e à complexidade dos regimes terapêuticos^(8,9)^. Segundo estudos, as taxas de adesão variam de 18,8% a 100%, com os imunossupressores apresentando índices ainda mais baixos, entre 31,3% e 88,8%^(8-10)^. Fatores como idade mais jovem, sofrimento psicossocial, apoio social e complexidade da terapia interferem na adesão^(8-10)^.

Apesar da disponibilidade de diversas tecnologias para auxílio na adesão à medicação, há desafios significativos no que diz respeito à implementação eficaz e à sustentabilidade de uso dessas ferramentas. Isso, possivelmente, ocorra em virtude da falta de envolvimento ativo dos usuários finais no processo de desenvolvimento, da escassez de fundamentos teóricos sólidos e da insuficiente atenção dedicada aos métodos de implementação por parte dos profissionais de saúde que poderiam viabilizar sua eficácia em cenários do cotidiano^(2)^.

Por fim, revisão sistemática recente identificou incipiência de estudos para avaliar como os fatores contextuais que influenciam a adesão se inter-relacionam e impactam a continuidade do tratamento no pós-TCTH^(10)^. Assim, o presente estudo se justifica pela inovação da análise de contexto proposta, que reside na utilização de uma estrutura de camadas interativas para compreensão ampla e integral do fenômeno. Acredita-se que essa abordagem permitirá a construção de ações mais eficazes para minimizar os desafios à adesão e melhorar a qualidade de vida dos pacientes pós-TCTH.

## OBJETIVOS

Analisar os contextos que influenciam a adesão ao tratamento farmacológico após a alta hospitalar de pacientes em fase de pós-transplante de células-tronco hematopoéticas.

## MÉTODOS

### Referencial teórico-metodológico

Trata-se de uma análise de contexto segundo a proposta de Hinds, Chaves e Cypress (1992), que objetiva elucidar o significado completo e compreender a totalidade de uma situação ou evento, de modo a permitir uma explicação ampla do fenômeno em questão.

O contexto é definido como uma estrutura composta por quatro camadas interativas: o imediato, o específico, o geral e o metacontexto. Essas camadas se distinguem entre si em três aspectos: quão amplamente o significado é compartilhado, desde a individualidade até um significado quase universal; tempo em que está focado, no presente ou no futuro; e velocidade com que a mudança ocorre e como é percebida em cada uma dessas camadas^(11)^.

Nessa perspectiva, o contexto imediato envolve a compreensão do fenômeno na forma em que ocorre. Já o contexto específico é moldado por várias circunstâncias do momento em questão, como fatores pessoais, ambientais e estado emocional. No contexto geral, são consideradas as interpretações desenvolvidas pelo indivíduo através das interações passadas e presentes. E, por último, o metacontexto é uma fonte de conhecimento socialmente construído que influencia diretamente os comportamentos e eventos^(11)^.

### Procedimentos metodológicos

Para operacionalização da análise de contexto, elaborou-se uma revisão de escopo de acordo com as diretrizes do JBI^(12)^, que seguiu as recomendações do *Preferred Reporting Items for Systematic reviews and Meta-Analyses extension for Scoping Reviews* (Prisma-ScR), com o protocolo de pesquisa registrado na plataforma *Open Science Framework* (OSF). Esse método tem por finalidade o mapeamento das evidências sobre determinada área, identificação de lacunas existentes, além de informar a necessidade de futuras investigações. A escolha por esse tipo de revisão para o presente estudo visa esclarecer o conceito estudado e identificar as principais características ou fatores a ele relacionados, conforme as diretrizes do JBI^(12)^.

Foram percorridas as etapas propostas por Peters et al.^(13)^: (1) Definição e alinhamento do objetivo e questão de pesquisa; (2) Desenvolvimento e alinhamento dos critérios de inclusão com o objetivo e questão de pesquisa; (3) Descrição da abordagem planejada para busca de evidências, seleção, extração de dados e apresentação das evidências; (4) Procura pelas evidências; (5) Seleção das evidências; (6) Extração das evidências; (7) Análise das evidências; (8) Apresentação dos resultados; e (9) Resumo das evidências em relação ao propósito da revisão.

Para elaboração da questão de pesquisa, utilizou-se o mnemônico PCC (P =População: Pacientes em fase de pós-TCTH; C = Conceito: Adesão ao tratamento farmacológico; C = Contexto: Alta hospitalar). Assim, estabeleceu-se a seguinte questão: “Quais os contextos que influenciam a adesão ao tratamento farmacológico após a alta hospitalar de pacientes em fase de pós-TCTH?”.

Inicialmente, foi realizada uma busca ampla na plataforma OSF e em bases de dados para a identificação de protocolos ou revisões com temática equivalente. A partir da não identificação de estudos similares, prosseguiu-se com as demais etapas.

A estratégia de busca foi dividida em duas fases. A primeira consistiu em uma pesquisa inicial nas bases de dados *National Library of Medicine* (PubMed) e *Cumulative Index to Nursing and Allied Health Literature* (CINAHL) para análise das palavras contidas nos títulos e resumos dos artigos recuperados que representassem o objeto de estudo. Essas palavras foram pesquisadas no *Medical Subject Headings* (MeSH)/Descritores em Ciências da Saúde (DeCS) e, em seguida, elencados os descritores controlados, a saber: *medication adherence*/adesão à medicação; *hematopoietic stem cell transplantation*/transplante de células tronco hematopoéticas; *stem cell transplantation*/transplante de células tronco; *bone marrow transplantation*/transplante de medula óssea. Também foram selecionadas as palavras-chave: *drug adherence*/adesão ao medicamento e *adherence*/adesão.

A segunda fase correspondeu a uma segunda busca em todas as fontes de dados selecionadas utilizando a seguinte estratégia: ((“*medication adherence*” OR “*drug adherence*” OR *adherence*) AND (“*hematopoietic stem cell transplantation*” OR “*stem cell transplantation*” OR “*bone marrow transplantation*”)). Salienta-se que, quando necessário, a estratégia empregada foi ajustada para se adequar às particularidades de cada fonte de dados.

### Fonte de dados

Para um mapeamento abrangente da literatura sobre o tema estudado foram selecionadas as seguintes bases de dados: Scopus, *Web of Science* (WoS), *National Library of Medicine* (PubMed), CINAHL, *ScienceDirect*, Literatura Latino-Americana e do Caribe em Ciências da Saúde (LILACS), *The Education Resources Information Center* (ERIC), *Academic Archive Online* (DIVA), Repositório Científico de Acesso Aberto de Portugal (RCAAP), *Theses Canadá* e banco de dissertações e teses da CAPES. A escolha por essas fontes de dados se justifica por permitirem acesso a uma variedade de evidências cientificas de alta relevância e qualidade, incluindo estudos primários e secundários, teses e dissertações, bem como literatura nacional e internacional. A diversidade de fontes de dados é crucial para garantir uma cobertura completa e representativa do campo de pesquisa^(12)^.

### Coleta e organização dos dados

A etapa de seleção das evidências ocorreu entre os meses de outubro e dezembro de 2023 e foi conduzida por dois revisores de forma independente e devidamente treinados quanto aos critérios de seleção para este estudo. Esse processo seguiu duas etapas distintas: uma triagem inicial, na qual os títulos e resumos foram avaliados, resultando na seleção dos estudos para a segunda triagem, em que os textos completos foram analisados. Em caso de divergências, consultou-se um terceiro revisor.

Para exportação, organização e triagem inicial das referências encontradas, além da identificação e remoção das duplicatas, utilizou-se o *software Rayyan* ^®^, uma ferramenta computacional gratuita desenvolvida pela *Qatar Foundation* ^(14)^.

Constituíram critérios de inclusão: estudos relacionados à adesão ao tratamento farmacológico durante o pós-TCTH com pacientes de qualquer idade e de ambos os sexos, disponíveis na íntegra e em qualquer idioma por meio do acesso via Comunidade Acadêmica Federada (CAFe) e sem delimitação temporal. Foram excluídos editoriais, cartas ao autor, artigos de opinião, protocolos, livros e capítulos de livros. Os estudos duplicados foram considerados apenas uma vez.

A estratégia de extração dos dados consistiu na coleta das seguintes variáveis: título, autoria, ano de publicação, país de realização do estudo, desenho metodológico, objetivos, resultados e descrição das principais descobertas que se relacionam com a adesão ao tratamento farmacológico no pós-TCTH. Para tanto, foi utilizada uma planilha no *software Excel Microsoft Office* ^®^.

### Análise dos dados

Utilizou-se o modelo de análise de contexto conforme o referencial teórico-metodológico adotado. Após a seleção e leitura integral dos estudos, as principais descobertas relacionadas à adesão ao tratamento farmacológico no pós-TCTH foram agrupadas com base em suas similaridades. Essas influências foram, então, categorizadas de acordo com o foco contextual que representam: imediato, específico, geral ou metacontexto. Tal análise permitiu uma interação entre as discussões de forma a propiciar a compreensão do todo. Os demais dados extraídos dos estudos selecionados foram analisados de forma descritiva.

## RESULTADOS

Foram incluídos na amostra 27 estudos^(4,5,8,10,15-37)^. A Figura 1 apresenta o fluxograma do processo de busca e seleção das evidências, incluindo identificação, triagem e inclusão dos estudos.

**Figura 1:** Fluxograma do processo de seleção dos estudos, 2024

Dos estudos identificados, todos são artigos, principalmente publicados em 2023, em um total de sete (25,9%). Os Estados Unidos da América se destacam em número, com 10 estudos (37%) e, na sequência, aparecem Alemanha, Brasil e a França, com três estudos cada (11,1%). Quanto ao tipo de abordagem metodológica, prevaleceram 12 estudos observacionais (44,4%). O Quadro 1 apresenta as demais características dos estudos incluídos na amostra.

**Quadro 1:** Caracterização dos estudos incluídos na amostra segundo título, ano, país, delineamento/número de participantes, intervenções e desfechos (N=27), 2024

Título	Ano País	Delineamento/Número de participantes	Intervenções	Desfechos
*Evaluation of a patient self-medication program in allogeneic hematopoietic stem cell transplantation* ^(4)^	2022 Canadá	Comparação prospectiva pré e pós-coorte n=51	Programa de Automedicação para pacientes *versus* educação convencional sobre medicamentos para alta.	As pontuações medianas de autoeficácia foram 38/39 no grupo de educação convencional *versus* 39/39 no grupo de Programa de Automedicação.
*Evaluation of adherence and clinical outcomes in patients undergoing allogeneic haematopoietic stem cell transplantation* ^(5)^	2020 Espanha	Estudo observacional n=46	Registros de dispensação do Serviço de Farmácia.	Elevada adesão ao tratamento profilático, variando entre 80,4% e 100,0%.
*Improving the transition of highly complex patients into the community: impact of a pharmacist in an allogeneic stem cell transplant (SCT) outpatient clinic* ^(8)^	2013 Austrália	Estudo de coorte prospectivo n=23	Consultas por farmacêutico clínico especializado.	Contribuição para melhorar o gerenciamento e a adesão à medicação.
*Medication Adherence among Allogeneic Haematopoietic Stem Cell Transplant Recipients: A Systematic Review* ^(10)^	2023 Itália	Revisão sistemática	Medidas subjetivas de adesão foram usadas com mais frequência (78,6%) até o momento.	A prevalência mediana de adesão medicamentosa foi de 61,8% e não diminuiu ao longo do tempo. A não adesão tem natureza multifatorial e requer modelos de cuidado multidisciplinares.
*Reciprocal associations between beliefs about medicines, health locus of control and adherence to immunosuppressive medication in allogeneic hematopoietic cell transplant patients: Findings from the ADE-TRAM study* ^(15)^	2023 Italia	Estudo observacional n=50	A adesão foi medida com a Escala de Autogestão de Medicamentos Imunossupressores.	Pacientes que acreditam que sua doença hematológica é causada por fatores externos têm menor probabilidade de aderir ao tratamento.
*Medication Adherence in Patients with Hematologic Malignancies Who Are Hematopoietic Stem Cell Transplantation Survivors: A Qualitative Study* ^(16)^	2023 Estados Unidos da América	Qualitativo e parte prospectivo longitudinal n=30	As entrevistas exploraram os fatores físicos, sociais, psicológicos e sociodemográficos que facilitam ou desencorajam a adesão ao regime de medicação pós-transplante.	Facilitadores e barreiras à adesão à medicação podem ser físicos, psicológicos, organizacionais e sociais.
*Recommendations for Providing Medication Adherence Support After Pediatric Hematopoietic Stem Cell Transplant: Caregivers’ Lived Experience* ^(17)^	2023 Estados Unidos da América	Estudo qualitativo n=29	Realizadas entrevistas qualitativas semiestruturadas sobre experiência e recomendações para melhorar o suporte à adesão à medicação fornecido pela equipe médica.	Os apoios práticos à adesão à medicação devem ser adaptados com base nas necessidades individuais da família.
*Adherence to Immunosuppressants among Adult Patients after Allogeneic Hematopoietic Stem-Cell Transplantation (Allo-HSCT): A Cross-Sectional Study* ^(18)^	2023 Irã	Estudo transversal n=110	Aplicação da antiga versão persa validada da Escala Morisky de Adesão à Medicação (MMAS-8) de oito itens.	A não adesão à medicação foi alta, o que indica a necessidade de monitoramento mais cuidadoso do uso de medicamentos
Orientações para o autocuidado de pacientes no pós-transplante de células-tronco hematopoéticas: revisão de escopo^(19)^	2023 Brasil	Revisão de escopo	Mapear as evidências sobre as orientações realizadas para o autocuidado de pacientes no pós-TCTH.	Seguir os cuidados relacionados às medicações é um dos maiores desafios. Esse fato tem relação com a não adesão à farmacoterapia, com o déficit de apoio social e com a necessidade de suporte instrumental.
*Impact of the insertion of the clinical pharmacist in the Allogeneic Hematopoietic Stem Cells Transplantation team* ^(20)^	2023 Brasil	Estudo intervencionista n=61	Acompanhamento farmacoterapêutico.	Aumento no conhecimento e na adesão.
*Can pharmacotherapeutic follow-up after allogeneic hematopoietic stem cell transplantation improve medication compliance?* ^(21)^	2023 Brasil	Estudo de intervenção n=27	Acompanhamento farmacoterapêutico.	Melhora na adesão à medicação e no conhecimento entre a primeira e a última consulta.
*Medication discharge teaching in pediatric hematopoietic stem cell transplantation: teaching characteristics, caregiver perceptions, and postdischarge adherence* ^(22)^	2022 Estados Unidos da América	Estudo qualitativo n=19	Ensinamentos de alta de medicamentos gravados em vídeo, conduzidos por farmacêuticos.	Os cuidadores relataram altos níveis de confiança em relação às suas capacidades de entender e gerenciar o regime de medicação de seus filhos.
*Rates and Predictors of Nonadherence to the Post-Allogeneic Hematopoietic Cell Transplantation Medical Regimen in Patients and Caregivers* ^(23)^	2022 Estados Unidos da América	Estudo prospectivo n=183	Versão modificada da Avaliação de Hábitos de Saúde.	11,2-15,7% da amostra relataram não adesão à medicação imunossupressora, 34,8-38,6% a outros tipos de medicamentos. Os preditores de não adesão mais consistentes foram as percepções do paciente.
*Exploring Stem Cell Transplanted Patients’ Perspectives on Medication Self-Management and Electronic Monitoring Devices Measuring Medication Adherence: A Qualitative Sub-Study of the Swiss SMILe Implementation Science Project* ^(24)^	2022 Suíça	Subestudo exploratório e qualitativo n=6	Modelo de Cuidado Integrado em transplante alogênico de células-tronco facilitado pela *e Health.*	Os participantes consideraram o *MEMS Button* um dispositivo viável para monitorar a adesão à medicação na vida cotidiana.
*Immunosuppression medication adherence after allogeneic hematopoietic stem cell transplant: Impact of a specialized clinical pharmacy program* ^(25)^	2021 França	Desenho intervencionista prospectivo n=61	Programa de farmácia clínica especializada.	Os níveis séricos na faixa alvo terapêutica foram maiores no grupo de intervenção (61,5% *versus* 53,0%)
*“This Graft-vs.-Host Disease Determines My Life. That’s It.”-A Qualitative Analysis of the Experiences and Needs of Allogenic Hematopoietic Stem Cells Transplantation Survivors in Germany* ^(26)^	2021 Alemanha	Estudo qualitativo n=40	Entrevistas semiestruturadas.	Participantes relataram não aderir aos cuidados prescritos, por exemplo, medicação ou isolamento, pois estavam sobrecarregados com a miríade de instruções.
*Psychosocial Pre-Transplant Screening With the Transplant Evaluation Rating Scale Contributes to Prediction of Survival After Hematopoietic Stem Cell Transplantation* ^(27)^	2021 Alemanha	Estudo prospectivo n=61	Triagens TERS (*Transplant Evaluation Rating Scale)* e MESI *(Medication Experience Scale for Immunosuppressants).*	A pontuação média de adesão de todos os 61 pacientes avaliados pelos médicos assistentes foi 2/5. A adesão do paciente correlaciona-se significativamente com as pontuações TERS.
*Medication non-adherence after allogeneic hematopoietic cell transplantation in adult and pediatric recipients: a cross sectional study conducted by the Francophone Society of Bone Marrow Transplantation and Cellular Therapy* ^(28)^	2021 França	Pesquisa multicêntrica transversal n=242	Teste de Avaliação da Conformidade.	Idade como o único fator relacionado à não adesão.
*Clinicians and patients perspectives on follow-up care and eHealth support after allogeneic hematopoietic stem cell transplantation: A mixed-methods contextual analysis as part of the SMILe study* ^(29)^	2020 Alemanha	Estudo de métodos mistos n=60	Modelo de Cuidados Crônicos aprimorado por *eHealth*; Grupos focais.	Os comportamentos de saúde que se beneficiariam do suporte incluem adesão à medicação, prática de atividade física e prevenção de infecções.
*A Prospective Survey of Outpatient Medication Adherence in Adult Allogeneic Hematopoietic Stem Cell Transplantation Patients* ^(30)^	2020 Estados Unidos da América	Estudo transversal n=200	Escala de Aderência à Medicação de Morisky e Escala de Aderência à Terapia Imunossupressora.	Metade (51%; 102 de 200) dos receptores de transplante alogênico relataram não adesão a medicamentos não imunossupressores.
*Standardized Semi-structured Psychosocial Evaluation before Hematopoietic Stem Cell Transplantation Predicts Patient Adherence to Post-Transplant Regimen* ^(31)^	2019 Estados Unidos da América	Estudo retrospectivo n=85	Aplicação da *Stanford Integrated Psychosocial Assessment for Transplantation*	O risco psicossocial quantificado correlacionou-se com a adesão ao regime pós-transplante.
*Medication adherence after pediatric allogeneic stem cell transplantation: Barriers and facilitators* ^(32)^	2018 França	Pesquisa qualitativa n=15	Entrevistas semiestruturadas foram conduzidas por um farmacêutico.	Necessidade dos cuidadores: levar em consideração a entidade familiar.
*Facilitators and Barriers to Self-Management for Adolescents and Young Adults Following a Hematopoietic Stem Cell Transplant* ^(33)^	2017 Estados Unidos da América	Pesquisa de teoria fundamentada n=30	Entrevista semiestruturada.	Os facilitadores incluíam ter uma atitude positiva, apoio social, organização, motivação e informação.
*Relationship between neurocognitive functioning and medication management ability over the first 6 months following allogeneic stem cell transplantation* ^(34)^	2016 Canadá	Estudo prospectivo n=58	Utilizados o *Medication management task* (revisado) e testes de funcionamento neurocognitivo.	Pacientes com comprometimento neurocognitivo eram mais propensos a ter capacidade de gerenciamento de medicamentos em uma tarefa simulada prejudicada.
*Adherence to outpatient oral medication regimens in adolescent hematopoietic stem cell transplant recipients* ^(35)^	2014 Estados Unidos da América	Estudo de intervenção n=6	Frascos eletrônicos de comprimidos *(Medical Event Monitors).*	As dificuldades de adesão diminuíram de 91% durante o primeiro mês após a alta para menos de 80% decorridos 3 meses e menos de 60% após 6 meses.
*Utilization of collaborative practice agreements between physicians and pharmacists as a mechanism to increase capacity to care for hematopoietic stem cell transplant recipients* ^(36)^	2013 Estados Unidos da América	Revisão	Utilização de farmacêuticos para administrar a terapia medicamentosa por meio de acordos de prática colaborativa.	Os farmacêuticos têm a base de conhecimento e as habilidades para auxiliar médicos e profissionais de prática avançada com o gerenciamento da terapia medicamentosa.
*Medication Adherence in Hematopoietic Stem Cell Transplantation: A Review of the Literature* ^(37)^	2017 Estados Unidos da América	Revisão da literatura	Os métodos para medir a adesão basearam-se no autorrelato.	A adesão diminuiu ao longo do tempo em todos os estudos, exceto num estudo de intervenção.

O Quadro 2 apresenta uma síntese das principais descobertas que se relacionam com a adesão ao tratamento farmacológico no pós-transplante de células-tronco hematopoéticas identificadas na amostra, além do agrupamento realizado de acordo com o eixo de discussão e contexto de análise conforme o método proposto por Hinds, Chaves e Cypress (1992).

**Quadro 2:** Síntese das principais descobertas que se relacionam com a adesão ao tratamento farmacológico no pós-transplante de células-tronco hematopoéticas identificadas na amostra (N=27), 2024

Eixo de discussão/Contexto de análise	Principais descobertas que se relacionam com a adesão ao tratamento farmacológico no pós-TCTH
Fatores intrínsecos ao paciente, como aspectos psicológicos, físicos e cognitivos/Contexto imediato	Aspectos psicológicos, como estresse, depressão, isolamento social, tensão, falta de motivação e perseverança, esquecimento e sofrimento moderado a grave interferem na adesão ao tratamento^(20,23,27-31)^.
	Descuido com a medicação pela piora dos sintomas^(30)^.
	Pacientes com comprometimento neurocognitivo estavam mais propensos a ter capacidade de gerenciamento de medicamentos prejudicada^(34)^.
	Pacientes com menor preocupação em relação aos medicamentos imunossupressores apresentaram maior probabilidade de aderência^(15)^.
Gerenciamento da medicação em domicílio/Contexto específico	Facilitadores para o gerenciamento da medicação: Experiência anterior com medicamentos^(16)^ Apoio de materiais de comunicação^(17)^ Intervenções: educação em saúde, caixa de medicamentos, simplificação do regime de medicação, lembretes, modificação de comportamento, apoio social, apoio e acompanhamento por prestadores de cuidados de saúde e incentivos de seguros^(8,22,26,33,37)^.
	Barreiras para o gerenciamento da medicação: Dificuldade no abastecimento de medicamentos e gerenciamento logístico de seguros^(16)^ Novas condições no domicílio^(18)^ Quanto ao tratamento: tempo de dose de medicação, complexidade do tratamento e mudanças frequentes dos medicamentos^(16,21,33)^ Sobrecarga de informações^(26)^ Tipo de transplante realizado^(31,36)^
Relação entre profissionais e pacientes/Contexto geral	Falta de educação adequada do paciente quanto ao uso de medicamentos interfere na adesão^(18)^.
	Os pacientes pós-TCTH devem estar cientes das orientações de autocuidado, possibilitando à equipe de enfermagem fornecer as informações necessárias para os cuidados domiciliares^(19)^.
	Supervisão de enfermagem na autoadministração de medicamentos dos pacientes^(10)^.
	Necessidade de boas interações entre paciente, cuidador e sistema de saúde para dar possibilidade e habilidade ao paciente de seguir o tratamento^(33)^.
	Alguns pacientes podem necessitar de esforços intensivos de intervenção antes e imediatamente após a alta^(36)^.
Diretrizes e programas institucionais/Metacontexto	O Programa de Automedicação para Pacientes está associado a um conhecimento duradouro e melhorado da medicação^(4)^.
	Diretrizes do Programa Nacional de Doadores de Medula Óssea^(23)^.
	Há déficits no programa de transplante do hospital em estudo referente ao preparo para alta, apoio ao autogerenciamento e cuidados de transição^(24)^.
	Programa de Farmácia Clínica Especializada pode ser benéfico para a adesão às drogas imunossupressoras^(25)^.

Após realizar o agrupamento e categorização por foco contextual, foram criados os seguintes subtemas:

Contexto imediato: aspectos psicológicos, físicos e cognitivos do paciente;

Contexto específico: facilitadores e barreiras para o gerenciamento da medicação em domicílio;

Contexto geral: particularidades da relação paciente-profissional;

Metacontexto: diretrizes e programas institucionais para o cuidado do paciente pós-TCTH.

Além disso, criou-se uma representação das camadas inter-relacionadas e suas principais características, conforme a Figura 2.

**Figura 2:** Camadas contextuais da adesão ao tratamento farmacológico no pós-transplante de células-tronco hematopoéticas, 2024

## DISCUSSÃO

### Contexto imediato: aspectos psicológicos, físicos e cognitivos do paciente

A eficácia do TCTH está intrinsecamente ligada à adesão rigorosa à terapia medicamentosa, que frequentemente requer a administração de diversos fármacos em horários e dosagens específicas. Contudo, a manutenção dessa adesão a longo prazo pode se tornar um desafio substancial, sobretudo por fatores associados ao paciente. Estes englobam alguns aspectos psicológicos, físicos e cognitivos que interferem diretamente na capacidade de seguir o tratamento conforme prescrito, de forma intencional ou não^(20,23,27-31,34)^.

Dentre estes, o sofrimento psicológico apresenta maior destaque, o que prejudica os cuidados em saúde. Depressão, estresse, isolamento social, tensão psicossocial, angústia e negação do tratamento foram os aspectos mais relatados em estudos^(15,20,27-29,31)^. O possível motivo desses achados é a preocupação com o prognóstico do transplante e as potenciais complicações na fase de recuperação^(38)^.

Esse achado fortalece as conclusões de uma pesquisa recente, segundo a qual as percepções subjetivas sobre a condição de saúde e o uso de medicamentos podem ter maior relevância na explicação do comportamento de adesão em comparação com as características não modificáveis, como idade e sexo^(39)^.

Além disso, as estratégias de enfrentamento individuais exercem influência na cooperação do paciente com o tratamento prescrito. Embora a variabilidade das estratégias de enfrentamento entre as pessoas dificulte definir o tipo de adaptação ideal durante o pós-transplante, é crucial ressaltar que, quando mal administradas, tais estratégias podem causar efeitos negativos e prejudiciais, como o surgimento de crises de ansiedade, depressão e menor adesão ao tratamento^(16,39)^.

Já os aspectos físicos estão relacionados aos efeitos colaterais dos medicamentos, como náuseas, fadiga e outros desconfortos. Esses sintomas podem variar de intensidade e frequência entre os pacientes e comprometer a motivação e disposição para adesão ao tratamento. Ademais, o próprio processo de transplante pode ser extremamente desgastante fisicamente, fato que torna ainda mais desafiador para o indivíduo manter-se comprometido com as terapias prescritas^(17)^.

Nesses momentos, é natural que o paciente se questione sobre a real eficácia do tratamento e se os desconfortos são suportáveis, principalmente pelo longo período de recuperação pós-transplante. Observa-se que os fatores físicos e psicológicos interagem de maneira complexa, pois podem contribuir para a banalização do tratamento e falta de motivação^(2)^.

Importante mencionar também que o paciente com comprometimento cognitivo pode ter dificuldade para se lembrar das orientações sobre o tratamento farmacológico, com consequente gerenciamento de medicamentos prejudicado^(30,34)^. Apesar de ser um desafio específico, cabe ressaltar a relação entre queixas cognitivas subjetivas e sofrimento psicológico, especialmente em situações conflitantes. Assim, é possível sugerir que o desempenho neuropsicológico não seja o principal motivo para o relato de problemas cognitivos^(40)^.

### Contexto específico: facilitadores e barreiras para o gerenciamento da medicação em domicílio

O gerenciamento do tratamento farmacológico após a alta hospitalar refere-se às medidas e práticas adotadas para garantir que os pacientes continuem a tomar os medicamentos prescritos de forma adequada no domicílio. Como facilitadores desse gerenciamento, destacam-se a rede de apoio e as intervenções de suporte^(16,17)^.

A inclusão de outras pessoas em uma rede de apoio, como familiares e amigos, é essencial para o cuidado centrado no paciente e se configura como o principal facilitador da adesão terapêutica. Contudo, para que esse apoio seja efetivo, faz-se necessário diferenciá-lo do total controle sobre as atividades diárias do paciente e encorajar que desenvolvam independência e autonomia^(41)^.

Nesse contexto, o papel de apoio pode abranger o enfrentamento das sequelas do transplante a longo prazo, auxílio para recordar as informações fornecidas pelos profissionais e orientação no gerenciamento dos cuidados após a alta^(32,37)^. Paralelamente, intervenções de suporte, somadas ao apoio social, também demonstraram facilitar a adesão, a exemplo de manter caixa organizadora de medicamentos, alarmes telefônicos e manuais educativos para minimizar confusões e esquecimentos^(16,20,37)^.

Conforme revisão sistemática, compreender a finalidade e a forma de uso de um medicamento é fundamental para que os indivíduos assumam a responsabilidade pelos seus tratamentos, tornando-se participantes engajados no processo de prescrição e administração destes fármacos^(42)^. Dessa forma, as intervenções devem ser inseridas em um contexto educacional maior, ou seja, devem complementar as orientações e demais cuidados da equipe de saúde^(21)^.

Por outro lado, com os avanços nas modalidades de tratamento, as taxas de sobrevida após o transplante aumentaram significativamente. No entanto, esse cenário trouxe consigo o desafio da autogestão das medicações prescritas por um longo período pós-transplante e, como resultado, a própria natureza da terapêutica tornou-se a principal barreira mencionada nos estudos abrangidos pela revisão^(5,37)^. O impacto desse desafio pode variar de acordo com o tipo de transplante realizado. Por exemplo, no caso do transplante autólogo em comparação com o alogênico, há menor tempo de hospitalização, acompanhamento menos intensivo e ausência de prescrição de imunossupressores a longo prazo^(31)^.

A dificuldade de acesso aos medicamentos foi outra barreira identificada, em grande parte pelo gerenciamento logístico dos seguros de saúde e reabastecimento tardio dos medicamentos pelas farmácias especializadas^(16)^. Metanálise revelou probabilidade 11,0% maior de não adesão aos medicamentos entre populações com seguro-saúde que exigem coparticipação para compra dos medicamentos. Essa falta de adesão pode elevar os custos para o sistema de saúde devido ao aumento das despesas com internações causadas pela não utilização de medicamentos essenciais^(43)^.

### Contexto geral: particularidades da relação paciente-profissional

As especificidades circunscritas na relação paciente-profissional influenciam diretamente a adesão ao tratamento, por ocasionarem obstáculos ou facilitarem o processo de autocuidado. Importante compreender que essa relação vai além das consultas médicas, pois abrange os demais profissionais envolvidos, especialmente a equipe de enfermagem, e inclui a abordagem do autocuidado do paciente submetido ao TCTH^(19)^.

No que se refere à equipe de saúde, a falta de coordenação entre os profissionais contribui para maior confusão em relação às orientações fornecidas aos pacientes e familiares^(32)^. Este fato pode ser considerado um reflexo do paradigma flexneriano que modelou as matrizes curriculares dos cursos de graduação na área da saúde, dado o enfoque nos aspectos biológicos e medicalizantes, sem uma visão integrada do paciente^(42)^.

Essa abordagem fragmentada pode prejudicar a qualidade e a continuidade do cuidado ao paciente no domicílio, de modo que uma comunicação positiva entre a própria equipe se torna um valioso aliado para proporcionar melhor adesão ao tratamento. Soma-se ao exposto a prática de uma abordagem sensível à singularidade de cada indivíduo como estratégia capaz de potencializar o autogerenciamento dos cuidados pelo paciente após o transplante^(44)^.

Ademais, o foco excessivo nos aspectos médicos foi apontado como outro obstáculo nessa relação, ou seja, a ênfase predominante na terapia farmacológica e cura da doença, por vezes em detrimento da atenção à autogestão do cuidado pelo paciente. De fato, novas tecnologias médicas têm facilitado as chances de cura, mas nem sempre aliviam o sofrimento dos pacientes que lidam com doenças que limitam a vida^(45)^.

Ainda, ao considerar o sofrimento psicológico como um fator para não adesão ao tratamento, torna-se necessário que o cuidado ao paciente transplantado supere a abordagem curativista. Fundamental ainda que a equipe de saúde saiba abordar as preocupações e medos dos pacientes, incentivando-os a expressá-los em relação ao tratamento. A ressignificação dos cuidados pós-TCTH pode colaborar para maior aceitação ao tratamento, na medida em que os pacientes passem a compreender as razões desses cuidados^(46)^.

### Metacontexto: diretrizes e programas institucionais para o cuidado do paciente pós-TCTH

O modelo médico tradicional assume que a eficácia do tratamento é suficiente para garantir a adesão do paciente, e isso pode negligenciar outros fatores importantes que também interferem nesta adesão^(39)^. Devido à hegemonia desse paradigma assistencial, as diretrizes encontradas na literatura têm se voltado ao profissional de saúde, e nota-se uma lacuna na pesquisa dedicada às melhores recomendações acerca da adesão pós-TCTH^(31,47)^.

Cabe salientar que, frequentemente, os pacientes não seguem as orientações de autocuidado por falta de compreensão sobre a importância dessas diretrizes para o tratamento^(19)^. De acordo com pesquisas realizadas com pacientes no Brasil e na Espanha, a elevada quantidade de informações incluídas nas instruções de alta muitas vezes deixa os pacientes transplantados com dúvidas e incertezas sobre os cuidados necessários em casa. Além disso, alguns afirmam não seguir orientações importantes, como o uso de protetor solar, preservativos durante relações sexuais e cuidados com a alimentação^(48)^.

Como consequência, observa-se a desarticulação entre o tratamento hospitalar e ambulatorial, com consequentes falhas na comunicação, troca inadequada de informações e monitoramento prejudicado após a alta hospitalar^(24,29)^. Essa situação poderia ser mitigada por meio de investimentos em infraestrutura e capacitação de equipes para adotar uma abordagem mais coordenada no atendimento, juntamente com a implementação de programas integrados de cuidados.

Nesse sentido, na presente amostra, foram identificados dois estudos para avaliar o impacto de programas de saúde para pacientes em pós-TCTH. Um deles é o Programa de Automedicação para Pacientes implementado em unidades de transplante no Canadá, no qual os pacientes com alta hospitalar programada recebem medicamentos em frascos rotulados, semelhantes aos disponibilizados em farmácias convencionais, o que permite a autoadministração supervisionada por um enfermeiro^(4)^. O referido programa esteve associado a um melhor conhecimento sobre a medicação após a alta, persistindo por três a cinco semanas, em comparação com a assistência anterior. A estratégia utilizada parte do pressuposto de que o conhecimento é particularmente vital nesta população, uma vez que a complexidade do regime medicamentoso pode causar confusão e, por conseguinte, comprometer a adesão^(4)^. Já o outro é o Programa de Farmácia Clínica Especializada implementado em uma instituição hospitalar na França, baseado em consultas farmacêuticas realizadas um dia antes da alta hospitalar, com duas e quatro semanas após a alta e, em seguida, uma vez por mês até completar 100 dias pós-TCTH. No momento da consulta, o paciente recebe orientações sobre a prescrição dos medicamentos, a fim de reforçar a importância da adesão e fornecer um cronograma personalizado para administração destes fármacos^(25)^.

Neste contexto, considera-se essencial que a equipe multiprofissional, especialmente o enfermeiro, implemente ações educativas eficazes para garantir que as orientações sobre cuidados e autocuidados a serem seguidos em casa após a alta hospitalar sejam compreendidas e aplicadas adequadamente. O enfermeiro desempenha um papel crucial nesse processo por sua proximidade com o paciente, seu papel educativo contínuo e sua capacidade de fornecer esclarecimentos detalhados. Isso não só facilita a adesão às orientações como melhora a eficácia dos cuidados domiciliares^(48)^.

### Limitações do estudo

Como limitação, destaca-se a escassez de estudos de intervenção na amostra, os quais podem fornecer uma compreensão mais robusta do impacto de contextos específicos na adesão à farmacoterapia.

### Contribuições para a Área da Enfermagem, Saúde ou Política Pública

A descrição dos diferentes contextos que influenciam a adesão ao tratamento farmacológico pós-TCTH amplia o conhecimento teórico da enfermagem e oferece aos profissionais orientações, baseadas em evidências científicas, para construção de intervenções de apoio e suporte ao tratamento. O estudo também proporciona uma compreensão da experiência do paciente, no sentido de facilitar uma assistência em saúde integral com enfoque em suas reais necessidades. Com tais direcionamentos, espera-se que a qualidade do cuidado pós-TCTH seja aprimorada, com incentivo ao desenvolvimento de outras pesquisas na área, a fim de ampliar ainda mais a temática.

## CONSIDERAÇÕES FINAIS

A adesão ao tratamento farmacológico pós-TCTH é influenciada por diferentes fatores contextuais inter-relacionados, que se manifestam por meio da complexa interação entre aspectos físicos, psicológicos, cognitivos e econômicos de cada paciente, bem como pela qualidade da relação estabelecida com os profissionais de saúde e presença de facilitadores e barreiras externas, nem sempre passíveis de modificação. Soma-se ao exposto o contexto global, que se refere às diretrizes e programas de saúde que desempenham papel crucial no suporte e na eficácia geral do tratamento.

Sugere-se a necessidade de pesquisas abrangentes sobre a adesão ao tratamento farmacológico pós-TCTH no Brasil, especialmente devido à diversidade cultural, socioeconômica e de acesso aos serviços de saúde entre as suas regiões que pode prejudicar significativamente a adesão. Para tanto, estudos multicêntricos são essenciais, pois permitem uma abordagem mais representativa pela inclusão de participantes de diferentes localidades do país.

## Data Availability

Não se aplica.
